# Neighborhood Socioeconomic Characteristics Associated with the COVID-19 Incidence in Elementary School Children: An Ecological Study in Osaka City, Japan

**DOI:** 10.3390/children10050822

**Published:** 2023-04-30

**Authors:** Kan Oishi, Takaaki Mori, Tomoki Nakaya, Kojiro Ishii

**Affiliations:** 1 Graduate School of Health and Sports Science, Doshisha University, 1–3, Tatara-Miyakodani, Kyotanabe 610-0394, Japan; 2 Japan Society for the Promotion of Sciences, Kojimachi Business Center Building, 5-3-1, Kojimachi, Chiyoda 102-0083, Japan; 3 Graduate School of Environmental Studies, Tohoku University, 468-1, Aramaki-Aoba, Aoba, Sendai 980-8572, Japan; 4 Faculty of Health and Sports Science, Doshisha University, 1-3, Tatara-Miyakodani, Kyotanabe 610-0394, Japan

**Keywords:** COVID-19, socioeconomic characteristics, elementary school children

## Abstract

We aimed to determine whether neighborhood socioeconomic characteristics are associated with the coronavirus disease 2019 (COVID-19) incidence in elementary school children and, if so, the associated characteristics. We obtained data on the number of infected children from 282 public elementary schools and the socioeconomic characteristics of each school district in Osaka City, Japan. We examined associations between these variables through negative binomial regression analyses. The proportion of employment in the wholesale and retail trade industry and the college graduation rate were significantly positively and negatively associated, respectively, with the total number of COVID-19-infected children. It was discovered that percentages of employment in the accommodation and food service industries in Wave 2, wholesale and retail trade industries after Wave 3, and healthcare and social assistance industries in Wave 5 were significantly positively associated with the number of infected children; likewise, the college graduation rate in Wave 5 was significantly negatively associated with the number of infected children. Our findings provide insight into the relevant and important areas of focus for public health policymakers and practitioners to ensure reduced disparities in COVID-19 infection rates.

## 1. Introduction

Coronavirus disease (COVID-19) is an infectious disease that was first reported at the end of 2019 in Wuhan, China. On 11 March 2020, the World Health Organization announced that COVID-19 had attained pandemic status [1]. As of February 2022, there were approximately 4 million confirmed cases of COVID-19 in Japan. Since then, the pandemic has shown no signs of abatement [2]. The rate of infection among children is also increasing; in Osaka Prefecture (the second largest prefecture in Japan), the proportion of infected 0–19-year-olds increased from approximately 5% in the first wave to >20% in the fifth wave [3].

The association between COVID-19 incidence and socioeconomic characteristics has been studied worldwide, and several systematic reviews and meta-analyses have been published [4,5,6,7,8]. Nevertheless, two issues remain to be addressed with regard to the evidence accumulated to date. The first issue is the age of the participants. In children, COVID-19 affects both health and daily activities. In Japan, children have been deprived of valuable daily experiences, such as school trips and vacations. A study by Yamaguchi et al. [9] suggested that the rights of children were restricted during the COVID-19 pandemic in Japan. However, only a few studies have focused on children due to the higher risks of infection, severe illness, and death in older people than in children. To our knowledge, only one study, conducted in Brazil, has examined the association of socioeconomic factors with the number of COVID-19 infections in children [10].

The second issue to be addressed in the accumulated evidence is the target country. According to a report by the Organisation for Economic Co-operation and Development (OECD) [11], the average relative poverty rate among children for the 36 OECD member countries was 13.0%, compared to 13.9% for Japan. The relative poverty rate indicates the percentage of persons living with less than 50% of the median equivalized disposable income. The results showed that even in Japan, where few racial and ethnic minorities exist, the children’s relative poverty rate is higher than in other countries. This may vary from the situation in many other countries where ethnic minorities are a major cause of inequality. Accordingly, the risk factors for COVID-19 in Japan may differ from those previously reported in other countries. Yoshikawa and Kawachi [12] performed the only study that explored the demographic and socioeconomic factors related to COVID-19 outcomes in Japan. Their results showed that the annual household income, welfare recipient rate, unemployment rate, smoking rate, obesity rate, percentage of employees in retail, transportation and restaurant industries, and household crowding were factors that were significantly associated with COVID-19 incidence and death. However, this study analyzed data reported up to February 2021 at the prefectural level. Additionally, this study did not target children, thus warranting the need for a study targeting children with more up-to-date data at a neighborhood level and involving more detailed regional classifications.

Therefore, this study aimed to determine whether neighborhood socioeconomic characteristics are associated with the number of COVID-19-infected children in Japanese elementary schools and, if so, which characteristics are associated.

We reported several neighborhood socioeconomic characteristics that were associated with the number of COVID-19-infected children among elementary school children. High percentages of employment in the wholesale and retail trade industries and low educational background were characteristics that were predominantly associated with a high number of infected children. Our findings may help provide insight into relevant and important areas of focus for public health policymakers and practitioners to ensure reduced disparities in COVID-19 infection rates.

## 2. Materials and Methods

This study was based on publicly available secondary databases provided by the national and local governments and the private sector. Hence, institutional review board approval and informed consent were not required.

### 2.1. Target

This study was conducted in Osaka City, one of the largest cities in Western Japan, with approximately 2.75 million residents in an area of 225.3 km^2^ [13]. In addition, this city has several deprivation neighborhoods with welfare benefit rates more than three times higher than the national rate [14]. According to the Ministry of Health, Labor and Welfare [15], 982 of the 3000 homeless people identified in designated cities in Japan in 2021 resided in Osaka City. 

### 2.2. Number of Children with COVID-19 and the Total Number of Children

Osaka City Hall [16] (2022) clarified the name of the public school, the period of closure, the status of the infected person (student or school personnel), and the number of infected persons when the school was closed owing to a student or school personnel being infected with COVID-19 from 1 June 2020. Using these data, we confirmed the number of infections among public elementary school students for the period from 1 June 2020 to 30 November 2021. In Japan, students between the ages of 5 and 12 are enrolled in elementary school. Using the data available at Osaka City Hall, we surveyed the number of children at all Osaka City municipal elementary schools during the fiscal year of 2021 [17].

### 2.3. Investigation of Neighborhood Socioeconomic Characteristics

In Japan, students can attend public schools in their residential areas; a set of such public schools is called a “school district”. Since elementary schools instruct children to avoid commuting to the school districts alone, these districts serve as both commuting and residential areas for school-going children. Therefore, school districts are frequently used to define the neighborhood in studies involving Japanese children [18,19].

We obtained data on the socioeconomic characteristics of each school district [20]. We gained the socioeconomic characteristics data from the secondary data available from the national and local governments and the private sector. For some socioeconomic characteristics, obtaining values for each school district was difficult. We have estimated the block (*cho-cho-aza*)-level values for such socioeconomic characteristics in the past by weighting them by the school district area, and we obtained useful findings [19]. In this study, we used this as a reference and prorate by area ratio. The details are described in the following sections. All spatial analyses were performed using a geographic information system (GIS; ArcGIS^®^ Pro 2.9.2, Esri Japan Corp., Tokyo, Japan).

### 2.4. Proportion of Individuals Employed in Industries with Frequent Close Contact with the Public

Based on a previous study [12], we calculated the proportion of individuals employed in industries with frequent close contact with the public: transportation and postal services, wholesale and retail trade, accommodation and food services, and health care and social assistance industries. The 2015 Population Census, published by the Statistics Bureau of Japan [21], surveyed the total number of workers and the number of workers in each industry in each block. We overlapped the number of workers in each industry at the block level with the school district polygon data and computed the number of workers in each industry by the ratio of the size of the overlapped area per size of each school district. The total number of workers in the same school district was also summed. Finally, the proportion of individuals employed in each industry in the school district was calculated using the following equation:

*([Number of workers in each industry]/[Total number of workers]) × 100*

### 2.5. College Graduation Rate

**Figure 1:**
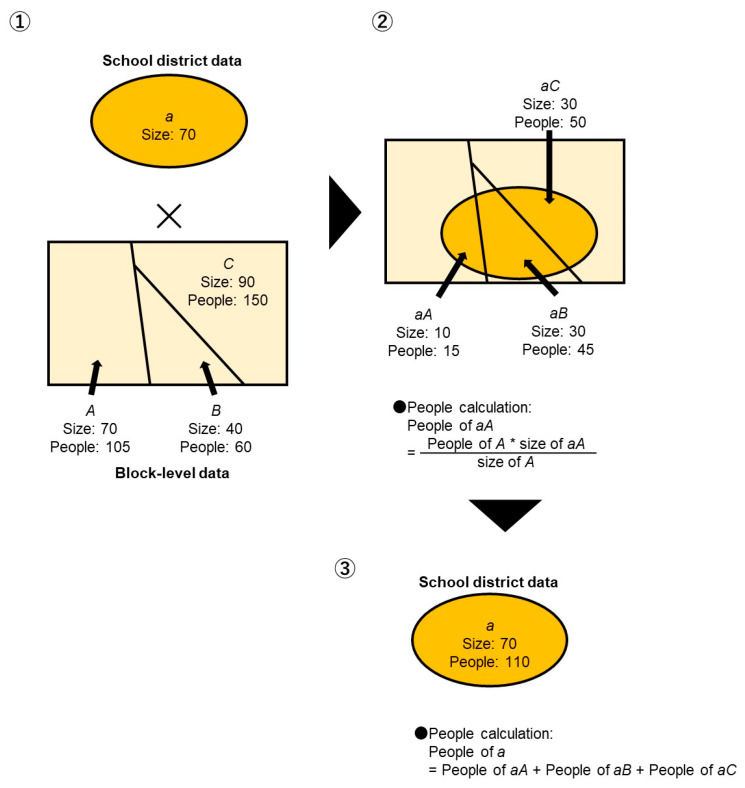
An example of the calculation of the number of people in each category (by each type of occupation and final education) for each school district.

In this study, the percentage of people aged ≥15 years, who graduated from a college or were post-undergraduate, was calculated from the national census data [22]. During the census, the total number of individuals, the number of individuals in each educational level, the number of those with an unknown education level, and in school-aged ≥15 years were surveyed for each block. In this study, we overlapped the population for each block-level category with the school district polygon data. Similar to the calculation of the proportion of individuals employed in industries that have close contact with the public, we overlapped the number of college or higher graduates, the total number of people aged ≥15 years, the number of individuals enrolled in school, and the number of individuals with no known educational background at their block-levels with the school district polygon data and computed the number in each category based on the ratio of the size of the overlapped area per size of each school district. Figure 1 shows an example of the calculation of the number of people in each occupation for each school district. 

Furthermore, the percentages of people who graduated from college or post-undergraduate in each school district were calculated using the following equation:

*([Number of college or higher graduates]/[Total number of people aged ≥ 15 years − Number of people enrolled in school − Number of people with no known educational background]) × 100*

### 2.6. Area Deprivation Index (ADI)

In this study, we employed the Japanese version of the ADI, which is an indirect measure of subjective and objective deprivation based on census items [23]. The details of the ADI have been previously published in a study by Nakaya et al. [24]. Several studies have produced useful findings regarding socioeconomic characteristics using the ADI [25,26]. The ADI was calculated at the block level using national census data [22]. We overlapped the block-level ADI and the number of households with school district polygon data [22]. Furthermore, we computed the number of households by the ratio of the size of the overlapped area per size of each school district. Finally, we calculated the weighted average of the ADI by the number of households and used that value as the ADI for the school district. The equation for calculating the weighted average is as follows:

$$ADIsd=\overset{n}{\underset{i=1}{\sum }}\left(Hi\cdot ADIi\right)\;/\overset{n}{\underset{i=1}{\sum }}Hi$$

**Figure 2:**
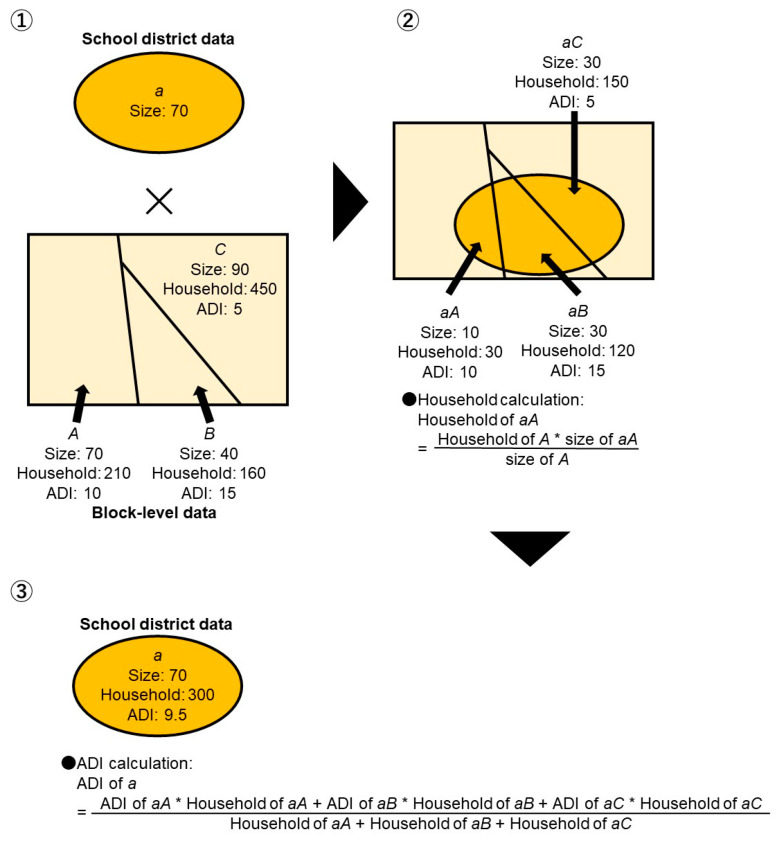
A sample calculation of the area deprivation index for each school district.

Here, “*sd*” indicates the school district and “*H*” indicates the number of households. Figure 2 shows a sample calculation of the ADI for each school district.

### 2.7. Covariates

Osaka City Hall [27] has published data on the population and number of households per school district based on data from the national census (Statistics Bureau of Japan, 2015). Thereafter, the population density (per km²) of a school district was calculated by dividing the population of that school district by the area of that school district. The number of people in the household was calculated by dividing the population by the number of households in each school district as well as the population density.

In addition, the points of stations, bus stops, and facilities (for older adults) that were based on the Digital National Land Information data, and the points of the address of medical treatment and testing institutions in Osaka City (as of 15 January 2022), based on the Osaka Prefectural Government website, were mapped on the GIS [28,29,30]. These points were then overlayed with the area data of the elementary school district, and the number of each type of facility in the elementary school district was tabulated. The number of each type of facility was then divided by the population of the corresponding school district in terms of the number of each type of facility per 1000 people in that school district.

### 2.8. Statistical Analysis

A negative binomial regression analysis was performed for the statistical analysis. The analysis was divided into four models according to the objectives. In all models, the objective variable was the number of COVID-19-infected children at the school level, and the offset term was the number of school children. In Model 1, we constructed a univariate model with one socioeconomic characteristic for each explanatory variable and no covariates. In Model 2, to remove the confounding effect of the demographic characteristics, we included one socioeconomic characteristic as an explanatory variable, and the population density, number of people in the household, number of public transportation modes, number of facilities for older adults, and number of medical treatment and testing institutions were included as covariates. In Model 3, to remove the confounding effects of the other socioeconomic characteristics, we simultaneously included all the socioeconomic characteristics as explanatory variables and did not include the demographic characteristics as covariates. Finally, in Model 4, to remove the confounding effect of all factors, we included all the socioeconomic characteristics as explanatory variables simultaneously, and the population density, number of people in the household, number of public transportation modes, number of facilities for older adults, number of households, number of public transport modes, and number of facilities for older adults were included as covariates. Table 1 presents the list of the objective variables, explanatory variables, covariates, and offset terms for all models.

The equation for the model of the negative binomial regression analysis is as follows:

$$\mathrm{Model}\;1:\;\log\left(\lambda_{i}\right)=\alpha +\beta_{i}x_{i}+\log\left(z_{i}\right)+\varepsilon_{i}$$

$$\mathrm{Model}\;2:\;\log\left(\lambda_{i}\right)=\alpha +\beta_{i}x_{i}+\overset{k}{\underset{i=1}{\sum }}(\gamma_{i}y_{i})+\log\left(z_{i}\right)+\varepsilon_{i}$$

$$\mathrm{Model}\;3:\;\log\left(\lambda_{i}\right)=\alpha +\overset{j}{\underset{i=1}{\sum }}\left(\beta_{i}x_{i}\right)+\log\left(z_{i}\right)+\varepsilon_{i}$$

$$\mathrm{Model}\;4:\;\log\left(\lambda_{i}\right)=\alpha +\overset{j}{\underset{i=1}{\sum }}\left(\beta_{i}x_{i}\right)+\overset{k}{\underset{i=1}{\sum }}(\gamma_{i}y_{i})+\log\left(z_{i}\right)+\varepsilon_{i}$$

Here, *λ* indicates the number of children with COVID-19, α indicates the intercept, *β* indicates the partial regression coefficients for each socioeconomic characteristic, *x* indicates each socioeconomic characteristic, γ indicates the partial regression coefficients for each covariate, *y* indicates each covariate, *z* indicates the number of students in the school, and ε indicates the error term.

**Figure 3:**
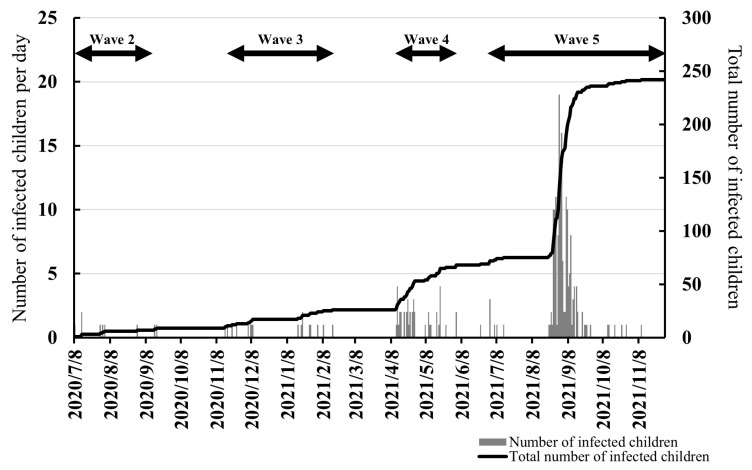
Changes in the number of infected children per day and the total number of infected children.

Four COVID-19 waves occurred in Japan during the survey period (Waves 2–5; Figure 3). Therefore, the number of infected children categorized by wave (Wave 2: 8 July 2020 to 17 September 2020; Wave 3: 15 November 2020 to 16 February 2021; Wave 4: 12 April 2021 to 3 June 2021; and Wave 5: 24 June 2021 to 10 November 2021) were also analyzed. Figure 3 shows the changes in the number of infected children per day and the total number of infected children in all target schools.

All statistical analyses were performed using R, version 4.2.2 (R Foundation for Statistical Computing, Vienna, Austria). A *p* < 0.05 was considered significant.

## 3. Results

The mean (standard deviation) number of COVID-19 cases, number of children, values of the socioeconomic variables, and values of the covariates for each school and school district are presented in Table 2.

According to our results, as depicted in Figure 3, the number of infected children per wave increased with each successive wave. Table 3 presents the incidence rate ratio (IRR) and 95% confidence interval (CI) of the negative binomial regression analyses.

In all analyses, the variance expansion coefficients for the explanatory variables were below 10. In all models for total waves, the proportion of the individuals employed in the wholesale and retail trade industries was significantly positively associated with the total number of infected children. In Model 3, the proportion of the individuals employed in the healthcare and social assistance industries was significantly positively associated with the total number of infected children, whereas in Models 3 and 4, the college graduation rate was significantly negatively associated with the number of infected children.

A wave-by-wave analysis was also conducted. In Models 2 and 4 of Wave 2, the percent of employment in the accommodation and food services industries showed a significant positive association with the number of infected children. In addition to Model 3 of Wave 2, Models 3 and 4 of Wave 5 showed that the college graduation rate was significantly negatively associated with the number of infected children. From Wave 3 onward, the proportion of individuals employed in the wholesale and retail trade industries was significantly positively associated with the number of infected children in all analyses except for Model 3 and 4 of Wave 3. In Model 1 of Wave 3, the ADI showed a significant negative association with the number of infected children. Finally, in the Wave 5 models, the proportion of the individuals employed in the healthcare and social assistance industries was significantly positively associated with the number of infected children. None of the socioeconomic characteristics were significantly associated with Wave 2, Model 1, and Wave 3, Model 3 and 4.

## 4. Discussion

We attempted to determine whether the neighborhood socioeconomic characteristics were associated with the number of COVID-19-infected children in Japanese elementary schools and, if so, we aimed to determine the associated characteristics. In Model 4, after adjusting for all the variables, individuals employed in the wholesale and retail trade industries were prominently associated with the number of children infected with COVID-19. A study in Utah showed that the wholesale trade industry reported the highest COVID-19 incidence compared to other workplace outbreak-associated cases in 13 industries [31]. A study comparing positivity rates by industry in Qatar also showed that the wholesale and retail trade sectors reported the highest positivity rate among 11 occupations [32]. Additionally, that study also described the retail industry as one of the industries with the highest risk of contracting COVID-19 in the workplace due to the nature of the job, which involves interacting with the public and focusing on the front lines. This study suggested that employment in the wholesale and retail trade industries in Japan is associated with a high risk of infection, which is also associated with the spread of infection among children living in the area.

In the wave-by-wave analysis, the proportion of individuals employed in the accommodation and food services industries showed a significant positive association with the number of infected children in Wave 2. In Japan, during the second wave of coronavirus that lasted from July to September 2020, the “Go To Travel” campaign was implemented, with the government covering 35% of the lodging costs for domestic travel and issuing locally usable coupons [33]. The campaign led to an increase in the number of infected people throughout Japan due to the increased number of migrants [34,35,36]. Thus, individuals employed in the accommodation and food services industries would have an increased risk of contracting COVID-19 infection during the campaign, leading to an increase in the number of infected children.

In Wave 5, the proportion of individuals employed in the healthcare and social assistance industries was significantly positively associated with the number of infected children. A study of more than 120,000 people in the United Kingdom showed that healthcare workers and social workers were seven times and approximately 2.5 times more at risk of severe COVID-19 compared with non-essential workers, respectively [37]. A study comparing COVID-19 incidence from Wave 1 to Wave 3 in Ontario, Canada, by industry, also found that individuals employed in the healthcare and social assistance industries were associated with the second highest incidence in Wave 1, the first highest incidence in Wave 2, and the third highest incidence in Wave 3 [38]. These findings suggest that employment in the healthcare and social assistance industries exposes individuals to a high risk of infection than in the aforementioned industries, leading to a high number of infected children.

The college graduation rate, a parameter for the measure of education, showed a significant negative association with the number of infected children in the total waves and Wave 5, whereas the ADI was not significantly associated with any wave. Hawkins et al. [39] also revealed that education was negatively associated with COVID-19 incidence in the United States. This could be due to the academic background not being considered a direct indicator of economic status but rather an indicator that reflects individuals’ knowledge, motivation and ability. A study on Japanese adults by Tokuda et al. [40] found that individuals with high health literacy scores were more likely to have a university or graduate school degree; however, this was not associated with household income. Therefore, the knowledge, willingness and ability of the community residents to obtain, understand, evaluate and use health-related information to adopt an infection prevention behavior, as opposed to the economic status of the community, could have an impact on the incidence of COVID-19 among Japanese children.

Finally, the significance of the associations of many socioeconomic characteristics changed between Models 1 and 4. To clarify whether the confounding factor was the demographic factors or other socioeconomic characteristics, we constructed Models 2 and 3. In Wave 2, employment in the accommodation and food services industry showed a significant positive association but only when adjusted for the demographic variables. The results indicated that demographic factors, such as population density, confounded these significant associations. The college graduation rate, however, showed significant associations only in models that take other socioeconomic characteristics into account. In other words, other socioeconomic characteristics confounded their significant associations. We did not identify a consistent pattern in the model with changing significance; however, we suggest that the demographic and other socioeconomic characteristics confounded the association between the socioeconomic characteristics and the number of COVID-19-infected children.

Martins-Filho et al. [10] was the only previous study to examine the association between socioeconomic characteristics and COVID-19 infection rates in children. However, their study found no significant association between the Social Vulnerability Index and the Gini coefficient being significantly associated with the infection rate, which was inconsistent with the present results. This difference may be explained by the study period, country/region and age group, as well as by the regional classification and socioeconomic characteristics. The previous study focused on incidence among 0–19 year-olds in Brazil until March 2020, whereas this study focused on incidence among 5–12 year-olds in Japan from June 2020 to November 2021. In addition, the regional classification in the previous study was by state, whereas this study used detailed school districts. Furthermore, while the previous study examined only economic indicators, this study also focused on education and occupation indicators. Therefore, future studies should examine the relationship among various socioeconomic characteristics in various countries, for various age groups, over a longer study period and by adopting detailed regional classifications.

This study had several limitations. First, multiple factors might have resulted in an underestimation of the number of infected children. For example, unconfirmed infections could have existed, as the students were asymptomatic and no tests were conducted. Furthermore, the data available on the Osaka City website were only for cases wherein an infection was confirmed and the schools were shut; therefore, it is possible that the data on infected people in schools that were not shut were unconfirmed. Furthermore, some children may not have been able to visit hospitals for certain reasons, such as financial reasons as well as the mentality of not wanting to inconvenience others; thus, the number of infected children could not be ascertained accurately. Second, the time frames in this survey, regarding the characteristics and the number of infected and enrolled children, were not consistent, and this gap could have led to differences in the results. Third, to calculate the number of people in each occupation, the college graduation rate, the ADI for each school district, the number of people in each category (by each type of occupation and final education), and the number of households in each school district were required. However, since these data could not be obtained for each school district, we used the estimated values obtained by dividing the block-level data proportionally by the area of the school district. This method was based on the assumption that the number of people and households in townships and streets is evenly distributed in relation to the area. However, the data may be unevenly distributed within the area, which may have an inaccuracy in the values. The fourth was about the goodness-of-fit of the equation in the results of this study, which was calculated and expressed by Akaike’s information criterion (AIC) (Table S1). The AIC was high for Model 2, which was adjusted for the demographic indicators (such as population density), and for Model 4, which was adjusted for the demographic indicators and other socioeconomic characteristics, and the equation did not fit well. However, even in Model 3, which had the lowest AIC in most cases, there was no significant change in the significance of the variables from the Model 4 results. Fifth, we analyzed the infection rates up to Wave 5 before the need to consider various additional factors in this study. This study used data before children ages 5 to 12 were vaccinated in Japan. In December 2021, the percentage of those with anti-severe acute respiratory syndrome coronavirus 2 (SARS-CoV-2) nucleocapsid (N) protein in Osaka Prefecture was 3.78%, and it is assumed that 96% of them did not have natural immunity [41]. It is unlikely that a failure to consider the vaccination and natural immunity acquisition rates in the model had a significant impact on the results of this study. On the other hand, as of 2023, 25% of Japanese children have received at least one vaccination [42], and the natural immunity acquisition rate was as high as 5.32% in Osaka Prefecture in February and March 2022 [43]. Several previous studies have reported that family socioeconomic characteristics are associated with COVID-19 infection in children [44,45,46]. This may make the disparity in COVID-19 in children even more pronounced. In addition, prior strains up to the Delta strains were prevalent during the period of this study. The route of COVID-19 infection among children was consistently from a home infection from the beginning. According to the database of the Japan Pediatric Society [47], the percentages of household infections among children in 2020 and 2021 were 69% and 71%, respectively. Therefore, it is unlikely that the emergence of mutant strains had a significant impact on the results of this study. On the other hand, the route of infection among children has changed since the SARS-CoV-2 Omicron strain became pandemic, with household infection accounting for 47% of all infections among children in 2022. It is undeniable that the results of this study may not be applicable to the current scenario due to changes in the route of infection caused by the emergence of the SARS-CoV-2 Omicron strains.

Despite these limitations, this was the first study to identify the socioeconomic characteristics associated with COVID-19 among elementary school children in Japan, a country with relatively homogeneous racial, ethnic and linguistic groups and specific disparities. Our findings may help provide insight into relevant and important focus areas for public health policymakers and practitioners to ensure reduced disparities in COVID-19 and other future infection rates for children. Specifically, we suggest that measures such as vaccination against COVID-19 infection and future infectious diseases need to be implemented while considering the socioeconomic characteristics, such as areas with many people with low educational backgrounds that reflect the meaning of health literacy as well as areas with many essential workers who communicate with others or have frequent contact with people at high risk of infection.

## 5. Conclusions

This study found that neighborhood socioeconomic characteristics were associated with the number of COVID-19 cases among elementary school children. In particular, high percentages of employment in the wholesale and retail trade industries and low educational backgrounds were prominently associated with a high number of infected children. Our findings suggest areas of focus for public health policymakers and practitioners to reduce the disparities in COVID-19 infection rates.

## Figures and Tables

**Table 1:** List of the objective variable, explanatory variables, covariates, and offset term for each model.

Model	Objective Variable	Explanatory Variable	Covariate	Offset Term
Model 1	The number of COVID-19-infected children	Input one socioeconomic characteristic at a time	None	The number of school children
Model 2	The number of COVID-19-infected children	Input one socioeconomic characteristic at a time	Adjusted for population density, number of people in the household, number of public transportation, number of facilities for older adults, and number of medical treatment and testing institutions	The number of school children
Model 3	The number of COVID-19-infected children	Input all socioeconomic characteristics simultaneously	None	The number of school children
Model 4	The number of COVID-19-infected children	Input all socioeconomic characteristics simultaneously	Adjusted for population density, number of people in the household, number of public transportation, number of facilities for older adults, and number of medical treatment and testing institutions	The number of school children

COVID-19: coronavirus disease 2019.

**Table 2:** Mean and standard deviation values of the number of COVID-19 cases, school population, socioeconomic characteristics, and covariates for each school and school district (N = 282).

Variables	Mean ± SD
Number of children with COVID-19 in Wave 2	0.03 ± 0.18
Number of children with COVID-19 in Wave 3	0.06 ± 0.25
Number of children with COVID-19 in Wave 4	0.15 ± 0.48
Number of children with COVID-19 in Wave 5	0.62 ± 0.92
Total number of children with COVID-19	0.86 ± 1.15
School population	402.01 ± 218.97
Employment in the transportation and postal services industry (%)	5.61 ± 2.63
Employment in the wholesale and retail trade industry (%)	16.17 ± 1.57
Employment in the accommodation and food services industry (%)	6.64 ± 1.21
Employment in the health care and social assistance industry (%)	10.74 ± 2.03
College graduation rate (%)	20.59 ± 8.31
ADI	6.79 ± 1.13
Population density (10,000 people/km^2^)	1.56 ± 0.62
Number of people in the household (people/family)	7.99 ± 9.36
Density of public transportation (/10,000 people)	1.94 ± 0.25
Density of facilities for older adults (/10,000 people)	1.19 ± 1.34
Density of medical treatment and testing institutions (/10,000 people)	1.22 ± 1.38

SD: standard deviation, COVID-19: coronavirus disease 2019, ADI: area deprivation index.

**Table 3:** Incidence rate ratios showing the association between the number of infected children and the neighborhood socioeconomic characteristics.

Explanatory Variables		Model 1 ^a^		Model 2 ^b^		Model 3 ^c^		Model 4 ^d^	
		IRR	(95% CI)	IRR	(95% CI)	IRR	(95% CI)	IRR	(95% CI)
Total	Employment in the transportation and postal services industry (%)	0.97	(0.91–1.04)	0.99	(0.92–1.07)	0.92	(0.84–1.01)	0.93	(0.84–1.03)
	Employment in the wholesale and retail trade industry (%)	1.22 ***	(1.11–1.35)	1.18 ***	(1.07–1.31)	1.19 ***	(1.08–1.31)	1.17 **	(1.06–1.29)
	Employment in the accommodation and food services industry (%)	1.03	(0.90–1.18)	1.10	(0.93–1.30)	1.11	(0.96–1.29)	1.06	(0.89–1.26)
	Employment in the health care and social assistance industry (%)	1.07	(1.00–1.15)	1.05	(0.97–1.14)	1.09 *	(1.01–1.18)	1.08	(0.99–1.18)
	College graduation rate (%)	0.99	(0.98–1.01)	0.98	(0.96–1.00)	0.95 *	(0.91–0.99)	0.95 *	(0.91–0.99)
	ADI	0.98	(0.85–1.12)	1.07	(0.91–1.25)	0.79	(0.59–1.05)	0.83	(0.59–1.14)
Wave 2	Employment in the transportation and postal services industry (%)	1.07	(0.81–1.34)	0.92	(0.65–1.23)	0.82	(0.54–1.17)	0.69	(0.41–1.06)
	Employment in the wholesale and retail trade industry (%)	0.86	(0.54–1.34)	0.91	(0.53–1.44)	0.89	(0.56–1.35)	0.91	(0.56–1.34)
	Employment in the accommodation and food services industry (%)	1.47	(0.88–2.17)	2.88 **	(1.44–5.88)	1.65	(0.91–2.99)	2.85 **	(1.33–6.43)
	Employment in the health care and social assistance industry (%)	0.89	(0.64–1.22)	0.83	(0.54–1.21)	0.98	(0.62–1.50)	0.89	(0.54–1.44)
	College graduation rate (%)	0.92	(0.83–1.01)	0.94	(0.83–1.04)	0.78 *	(0.60–0.98)	0.90	(0.68–1.13)
	ADI	1.33	(0.75–2.00)	1.33	(0.63–2.51)	0.37	(0.09–1.24)	0.80	(0.16–3.04)
Wave 3	Employment in the transportation and postal services industry (%)	1.05	(0.85–1.24)	1.08	(0.86–1.30)	1.27	(0.97–1.60)	1.22	(0.89–1.64)
	Employment in the wholesale and retail trade industry (%)	1.35 *	(0.99–1.80)	1.39 *	(1.01–1.85)	1.28	(0.96–1.68)	1.32	(0.97–1.76)
	Employment in the accommodation and food services industry (%)	1.07	(0.68–1.57)	1.03	(0.59–1.68)	1.02	(0.60–1.70)	0.99	(0.54–1.71)
	Employment in the health care and social assistance industry (%)	0.81	(0.63–1.02)	0.76	(0.56–1.01)	0.89	(0.67–1.15)	0.90	(0.63–1.24)
	College graduation rate (%)	1.04	(0.99–1.09)	1.02	(0.96–1.08)	1.01	(0.89–1.14)	1.00	(0.85–1.16)
	ADI	0.53 *	(0.30–0.88)	0.61	(0.31–1.12)	0.41	(0.15–1.16)	0.47	(0.10–1.89)
Wave 4	Employment in the transportation and postal services industry (%)	0.94	(0.78–1.12)	0.98	(0.78–1.20)	0.81	(0.61–1.04)	0.83	(0.61–1.09)
	Employment in the wholesale and retail trade industry (%)	1.38 **	(1.06–1.85)	1.34 *	(1.03–1.79)	1.41 **	(1.07–1.90)	1.34 *	(1.02–1.80)
	Employment in the accommodation and food services industry (%)	1.18	(0.83–1.65)	1.16	(0.76–1.76)	1.15	(0.78–1.68)	1.05	(0.66–1.63)
	Employment in the health care and social assistance industry (%)	0.97	(0.57–1.19)	0.98	(0.79–1.23)	0.97	(0.78–1.20)	1.00	(0.78–1.27)
	College graduation rate (%)	0.98	(0.93–1.03)	0.96	(0.91–1.02)	0.92	(0.82–1.03)	0.91	(0.80–1.03)
	ADI	1.05	(0.73–1.46)	1.09	(0.74–1.58)	0.92	(0.43–1.75)	0.86	(0.37–1.77)
Wave 5	Employment in the transportation and postal services industry (%)	0.97	(0.90–1.04)	0.98	(0.90–1.07)	0.91	(0.82–1.01)	0.94	(0.83–1.05)
	Employment in the wholesale and retail trade industry (%)	1.19 **	(1.06–1.33)	1.16 **	(1.03–1.30)	1.16 **	(1.04–1.29)	1.15 *	(1.03–1.29)
	Employment in the accommodation and food services industry (%)	0.98	(0.83–1.13)	1.04	(0.85–1.26)	1.08	(0.91–1.29)	1.02	(0.83–1.25)
	Employment in the health care and social assistance industry (%)	1.13 **	(1.04–1.22)	1.12 *	(1.02–1.22)	1.15 **	(1.05–1.25)	1.16 **	(1.05–1.28)
	College graduation rate (%)	0.99	(0.97–1.01)	0.98	(0.96–1.01)	0.95 *	(0.91–1.00)	0.94 *	(0.90–0.99)
	ADI	0.99	(0.85–1.16)	1.08	(0.90–1.29)	0.80	(0.57–1.11)	0.80	(0.54–1.16)

^a^: Univariate regression analysis; ^b^: multivariate regression analysis controlling for population density, number of people in the household, number of public transportation, number of facilities for older adults, and number of medical treatment and testing institutions; ^c^: multivariate regression analysis controlling for other socioeconomic characteristics; ^d^: multivariate regression analysis controlling for population density, number of people in the household, number of public transportation, number of facilities for older adults, number of medical treatment, testing institutions, and other socioeconomic characteristics; IRR: incidence rate ratio, CI: confidence interval; ADI: area deprivation index, *: *p* < 0.05, **: *p* < 0.01, ***: *p* < 0.001.

## Data Availability

The datasets analyzed during the current study are not publicly available because the publication of datasets that may identify schools is prohibited in Japan; however, the datasets are available from the corresponding author upon reasonable request.

## Supplementary Materials

The following supporting information can be downloaded at: https://www.mdpi.com/article/10.3390/children10050822/s1, Table S1: Akaike’s Information Criterion (AIC) for each regression equation.

## References

[1] World Health Organization (2020). WHO Director-General’s Opening Remarks at the Media Briefing on COVID-19. https://www.who.int/director-general/speeches/detail/who-director-general-s-opening-remarks-at-the-media-briefing-on-covid-19---11-march-2020.

[2] (2022). Ministry of Health, Labour and Welfare, Visualizing the Data: Information on COVID-19 Infections. https://covid19.mhlw.go.jp/en/.

[3] Osaka Prefectural Center for Infectious Diseases, COVID-19. http://www.iph.pref.osaka.jp/infection/disease/corona.html.

[4] Barceló M.A., Saez M. (2021). Methodological Limitations in Studies Assessing the Effects of Environmental and Socioeconomic Variables on the Spread of COVID-19: A Systematic Review. Environ. Sci. Eur..

[5] Magesh S., John D., Li W.T., Li Y., Mattingly-app A., Jain S., Chang E.Y., Ongkeko W.M. (2021). Disparities in COVID-19 Outcomes by Race, Ethnicity, and Socioeconomic Status. JAMA Netw. Open.

[6] Khanijahani A., Iezadi S., Gholipour K., Azami-Aghdash S., Naghibi D. (2021). A systematic review of racial/ethnic and socioeconomic disparities in COVID-19. Int. J. Equity Health.

[7] Benita F., Rebollar-Ruelas L., Gaytán-Alfaro E.D. (2022). What Have We Learned about Socioeconomic Inequalities in the Spread of COVID-19? A Systematic Review. Sustain. Cities Soc..

[8] Alidadi M., Sharifi A. (2022). Effects of the Built Environment and Human Factors on the Spread of COVID-19: A Systematic Literature Review. Sci. Total Environ..

[9] Yamaguchi A., Hosozawa M., Hasegawa A., Okubo Y., Sampei M., Sawada N., Piedvache A., Morisaki N., Hangai M., Tanaka K. (2021). The Coronavirus Disease 2019 Pandemic and the Rights of the Child in Japan. Pediatr. Int..

[10] Martins-Filho P.R., Quintans-Júnior L.J., de Souza Araújo A.A., Sposato K.B., Souza Tavares C.S., Gurgel R.Q., Fontes Leite D.C., de Paiva S.M., Santos H.P., Santos V.S. (2021). Socio-Economic Inequalities and COVID-19 Incidence and Mortality in Brazilian Children: A Nationwide Register-Based Study. Public Health.

[11] Society at a Glance 2019: OECD Social Indicators—OECD iLibrary. https://www.oecd-ilibrary.org/docserver/soc_glance-2019-en.pdf?expires=1555274574&id=id&accname=guest&checksum=638B78794EFD743B54BF58C7707D0038.

[12] Yoshikawa Y., Kawachi I. (2021). Association of Socioeconomic Characteristics with Disparities in COVID-19 Outcomes in Japan. JAMA Netw. Open.

[13] (2023). Osaka City Hall, Estimated Population (as of the 1st of Each Month) and Population Change. https://www.city.osaka.lg.jp/toshikeikaku/page/0000541634.html/.

[14] Osaka City Hall, Status of Application of Public Assistance, etc. https://www.city.osaka.lg.jp/fukushi/page/0000086901.html/.

[15] Ministry of Health, Labor and Welfare, Results of the National Survey on the Actual Conditions of the Homeless (Approximate Survey). https://www.mhlw.go.jp/content/12003000/000769666.pdf/2021.

[16] (2022). Osaka City Hall, Suspended Classes at Schools Due to Novel Coronavirus Infection. https://www.city.osaka.lg.jp/kyoiku/page/0000509375.html/.

[17] Osaka City Hall, Fiscal 2020 School Status Survey (as of 1 May 2021). https://www.city.osaka.lg.jp/kyoiku/page/0000511944.html/.

[18] Oishi K., Aoki T., Harada T., Tanaka C., Tanaka S., Tanaka H., Fukuda K., Kamikawa Y., Tsuji N., Komura K. (2021). Association of Neighborhood Food Environment and Physical Activity Environment with Obesity: A Large-Scale Cross-Sectional Study of Fifth- to Ninth-Grade Children in Japan. INQUIRY J. Health Care Organ. Provis. Financ..

[19] Mori T., Aoki T., Oishi K., Harada T., Tanaka C., Tanaka S., Tanaka H., Fukuda K., Kamikawa Y., Tsuji N. (2022). Neighborhood-Level Socioeconomic Factors Moderate the Association between Physical Activity and Relative Age Effect: A Cross-Sectional Survey Study with Japanese Adolescents. BMC Public Health.

[20] Geo-K L.L.C. (2018). School District (Elementary and Junior High School District Polygon and Point Database). https://www.gisdata-store.biz/product/1264/.

[21] (2015). Statistics Bureau of Japan, Population Census. https://www.e-stat.go.jp/en/stat-search/files?page=1&toukei=00200521&tstat=000001080615/.

[22] (2010). Statistics Bureau of Japan, Population Census. https://www.e-stat.go.jp/en/stat-search/files?page=1&toukei=00200521&tstat=000001039448/.

[23] Nakaya T. (2011). Evaluating socio-economic inequalities in cancer mortality by using areal statistics in Japan: A note on the relation between municipal cancer mortality and areal deprivation index. Proc. Inst. Stat. Math..

[24] Nakaya T., Honjo K., Hanibuchi T., Ikeda A., Iso H., Inoue M., Sawada N., Tsugane S. (2014). Associations of All-Cause Mortality with Census-Based Neighbourhood Deprivation and Population Density in Japan: A Multilevel Survival Analysis. PLoS ONE.

[25] Okubo R., Yoshioka T., Nakaya T., Hanibuchi T., Okano H., Ikezawa S., Tsuno K., Murayama H., Tabuchi T. (2021). Urbanization level and neighborhood deprivation, not COVID-19 case numbers by residence area, are associated with severe psychological distress and new-onset suicidal ideation during the COVID-19 pandemic. J. Affect. Disord..

[26] Kataoka A., Fukui K., Sato T., Kikuchi H., Inoue S., Kondo N., Nakaya T., Ito Y. (2021). Geographical socioeconomic inequalities in healthy life expectancy in Japan, 2010-2014: An ecological study. Lancet Reg. Health West Pac..

[27] (2016). Osaka City Hall, 2016 Census Results by Elementary District. https://www.city.osaka.lg.jp/toshikeikaku/page/0000341916.html/.

[28] Ministry of Land, Infrastructure, Transport and Tourism, National Land Information Download Service. https://nlftp.mlit.go.jp/ksj/gml/datalist/KsjTmplt-P11.html/.

[29] Ministry of Land, Infrastructure, Transport and Tourism, National Land Information Download Service. https://nlftp.mlit.go.jp/ksj/gml/datalist/KsjTmplt-N05-v1_3.html/.

[30] (2022). Osaka Prefectural Government, List of Medical Treatment and Testing Institution (All Medical Institutions). https://pref-osaka.viewer.kintoneapp.com/public/0513c2014b31535d76ce0b6d4a6516e1399e3ae432a81f7b1c400569491589dc#/.

[31] Bui D.P., McCaffrey K., Friedrichs M., LaCross N., Lewis N.M., Sage K., Barbeau B., Vilven D., Rose C., Braby S. (2020). Racial and Ethnic Disparities among COVID-19 Cases in Workplace Outbreaks by Industry Sector—Utah, 6 March 6–5 June 2020. MMWR Morb. Mortal. Wkly. Rep..

[32] Al-Kuwari M.G., Al-Nuaimi A.A., Abdulmajeed J., Semaan S., Al-Romaihi H.E., Kandy M.C., Swamy S. (2021). COVID-19 Infection across Workplace Settings in Qatar: A Comparison of COVID-19 Positivity Rates of Screened Workers from March 1st until July 31st, 2020. J. Occup. Med. Toxicol..

[33] (2020). Japan Tourism Agency, Go to Travel Campaign. https://www.e-stat.go.jp/stat-search?page=1&toukei=00601020&kikan=00601.

[34] Anzai A., Nishiura H. (2021). “Go To Travel” Campaign and Travel-Associated Coronavirus Disease 2019 Cases: A Descriptive Analysis, July–August 2020. J. Clin. Med..

[35] Kanamori R., Kawakami Y., Nojiri S., Miyazawa S., Kuroki M., Nishizaki Y. (2022). Changes in social environment due to the state of emergency and Go To campaign during the COVID-19 pandemic in Japan: An ecological study. PLoS ONE.

[36] Uchida M. (2021). Changes in numbers of COVID-19 cases among residents of sightseeing resort areas before and during the “Go to Travel” campaign: Descriptive epidemiology in Gunma Prefecture. Jpn. J. Infect. Dis..

[37] Mutambudzi M., Niedwiedz C., Macdonald E.B., Leyland A., Mair F., Anderson J., Celis-Morales C., Cleland J., Forbes J., Gill J. (2020). Occupation and risk of severe COVID-19: Prospective cohort study of 120 075 UK Biobank participants. Occup. Environ. Med..

[38] Buchan S.A., Smith P.M., Warren C., Murti M., Mustard C., Kim J.H., Menon S., Brown K.A., van Ingen T., Smith B.T. (2022). Incidence of outbreak-associated COVID-19 cases by industry in Ontario, Canada, 1 April 2020–31 March 2021. Occup. Environ. Med..

[39] Hawkins R.B., Charles E.J., Mehaffey J.H. (2020). Socio-economic status and COVID-19–related cases and fatalities. Public Health.

[40] Tokuda Y., Doba N., Butler J.P., Paasche-Orlow M.K. (2009). Health literacy and physical and psychological wellbeing in Japanese adults. Patient Educ. Couns..

[41] (2022). Ministry of Health, Labor and Welfare, Preliminary Results of the 3rd Antibody Retention Survey. https://www.mhlw.go.jp/content/10900000/000898612.pdf/.

[42] (2023). Prime Minister’s Office of Japan and His Cabinet, COVID-19 Vaccines. https://www.kantei.go.jp/jp/headline/kansensho/vaccine.html/.

[43] (2022). Ministry of Health, Labor and Welfare, Preliminary Results of the 4th Antibody Retention Survey. https://www.mhlw.go.jp/content/000945078.pdf/.

[44] Fazel M., Puntis S., White S.R., Townsend A., Mansfield K.L., Viner R., Herring J., Polland A.J., Freeman D. (2021). Willingness of children and adolescents to have a COVID-19 vaccination: Results of a large whole schools survey in England. EClinicalMedicine.

[45] Scharff A.Z., Paulsen M., Schaefer P., Tanisik F., Sugianto R.I., Stanislawski N., Blume H., Schmidt B.M.W., Heiden S., Stiesch M. (2022). Students’ age and parental level of education influence COVID-19 vaccination hesitancy. Eur. J. Pediatr..

[46] Singh G.K., Lee H., Azuine R.E. (2022). Marked disparities in COVID-19 vaccination among US children and adolescents by racial/ethnic, socioeconomic, geographic, and health characteristics, United States, December 2021–April 2022. Int. J. MCH AIDS.

[47] (2022). Japan Pediatric Society, COVID-19 pediatric Cases in Japan. https://www.coreregistry.jp/CoreRegistry_COVID19_CRF_Dashboard/Home/DashBoardviewer/.

